# Impact of pathological T3a upstaging on oncological outcomes of clinical T1 renal cell carcinoma: a meta-analysis

**DOI:** 10.7150/jca.32859

**Published:** 2019-08-27

**Authors:** Luyao Chen, Wen Deng, Xiaoqiang Liu, Gongxian Wang, Bin Fu

**Affiliations:** Department of Urology, The First Affiliated Hospital of Nanchang University, Nanchang, China

**Keywords:** T3a, upstaging, renal cell carcinoma, survival, meta-analysis

## Abstract

**Objective**: The study aims to assess the prognostic impact of pathological T3a upstaging in clinical T1 renal cell carcinoma (RCC) on clinical outcomes.

**Methods**: We performed a systematic literature search of PMC, Embase, Web of Science, and Cochrane library from inception to April 2019 for studies that investigated the prognostic significance of pathological T3a upstaging in clinical T1 RCC after surgery and conducted a standard meta-analysis on survival outcomes.

**Results**: Overall, nine studies including 101,505 clinical T1 RCC patients were identified, in which 5,560 (5.5%) patients were upstaged to T3a after surgical treatment. Meta-analysis results showed that pT3a upstaging from clinical T1 RCC was significantly associated with poor recurrence-free survival (RFS; pooled hazard ratio [HR] 2.16, 95% confidence interval [CI] 1.70-2.75; P<0.001), overall survival (OS; pooled HR 1.36, 95% CI 1.24-1.50; P<0.001), and cancer-specific survival (CSS; pooled HR 2.11, 95% CI 1.58-2.83; P<0.001). Subgroup analyses by surgical type demonstrated that pT3a upstaging remains a significant prognostic factor for RFS and OS in RCC patients who underwent different surgical treatments.

**Conclusions**: Current available evidence strongly supported that postoperative pT3a upstaging has a significant negative impact on RFS, OS, and CSS in clinical T1 RCC patients. Clinical T1 RCC patients with pT3a upstaging after surgery should be closely monitored by clinician and should receive close follow-up for their poor prognosis.

## 1. Introduction

Renal cell carcinoma (RCC), the third most common urological malignancy worldwide, accounts for approximately 3% of all reported cancers 1. Its incidence continues to steadily increase in most countries and regions 2. In the United States, approximately 65,340 new cases are diagnosed and 14,970 RCC-related deaths occur annually 3. The widespread use of non-invasive radiologic imaging tools including computed tomography (CT) and ultrasonography results in the overall increase in the incidental detection of RCC, especially small renal mass 4, 5.

Currently, clinical T stage is considered to be the most important factor for treatment decision-making of a renal mass and partial nephrectomy (PN) has been recommended as the first option for the surgical treatment of T1 RCC, because it preserves renal function and provides equivalent survival outcomes that are comparable to those of radical nephrectomy (RN) 6, 7. The widespread adoption of robotic surgery further extends the indication of PN for some complex T1 cases 8, 9. However, microscopic perirenal fat invasion, renal sinus fat infiltration, and renal vein invasion can be missed on preoperative CT 10, 11, and thus some cases appear to be clinical T1 (cT1) pre-operation but are actually pathological T3a (pT3a) post-operation owing to extension into fat or renal vein.

This interesting clinical issue (cT1 upstaged to pT3a) has attracted extensive attention and the prognosis of patients with pT3a RCC that is upstaged from a small renal mass (cT1) remains controversial. Some recent studies have focused on the prognostic significance of pT3a upstaging in cT1 RCC undergoing nephrectomy and suggested inconsistent results. Lee et al.^12^ demonstrated that patients with RCC upstaged from clinical stage T1 to pathologic stage T3a showed shorter survival outcomes than those without upstaging. Jeong et al. 13 found that pathological T3a upstaging of cT1 RCC was associated with a poor recurrence-free survival, compared with pT1 disease. Nayak's study 14 had similar conclusion and highlighted the importance of accurate clinical staging. On the contrary, Lee's study 15 suggested that pT3a stage disease after PN for small RCCs had similar oncological outcomes to those of pT1a stage disease. Moreover, the studies by Ramaswamy et al. 16 and Roberts et al. 17 revealed that pathologic upstaging did not result in worsened oncological outcomes after an intermediate follow-up.

In light of these conflicting findings in previous studies, we conducted a systematic review of published relevant studies and carried out a standard meta-analysis of extracted data that can be merged to evaluate of the oncological outcomes precisely in cases upstaged from cT1 to pT3a RCC after nephrectomy.

## 2. Methods

This study was performed according to the guideline of Preferred Reporting Items for Systematic Reviews and Meta-Analyses (PRISMA)^18^. Because the data were collected from published literatures, ethical approval was not needed.

### 2.1 Search strategy

A computerized bibliographic search of Electronic databases (PMC, Embase, Cochrane library and Web of Science) were carried out up to April 2019 to identify published studies investigating the prognostic significance of pT3a upstaging in cT1 RCC after surgery.

Search terms using MeSH headings, keywords, and text words consist of “upstage” or “upstaging” or “upstaged” combined with “renal tumor” or “renal cancer” or “renal cell carcinoma” or “kidney cancer”. Besides, the references cited in the relevant studies were also reviewed for possible inclusions. No language limitation existed in this process. The preliminary evaluation of identified studies were performed independently by two authors (Chen and Deng).

### 2.2 Inclusion and exclusion criteria

Inclusion criteria in our meta-analysis were literatures that confirmed the upstaging (cT1 upstaged to pT3a) by postoperative pathological examination and evaluated the impact of upstaging (cT1 upstaged to pT3a) on oncologic outcomes in cT1 RCC patients. The endpoints of oncologic outcomes included recurrence-free survival (RFS), cancer-specific survival (CSS) and overall survival (OS).

Exclusion criteria were listed as follows: (1) basic research; (2) non-original articles (letters, conference abstract, editorials, comments or review articles); (3) studies not focusing on cT1 RCC; (4) studies that discussed other forms of upstaging (cT1 upstaged to pT2 or cT2 upstaged to pT3a); (5) studies that did not provide oncologic outcomes with hazard ratio (HR) and corresponding 95% confidence interval (CI) or lacked sufficient data to achieve an estimated HRs and 95% CIs by using the methods reported by Tierney et al^19^.

When two or more published papers by the same authors were screened, the most informative article was selected to avoid incorporating duplicated data. Two authors (Chen and Deng) independently completed the review of titles, abstracts and full-text studies. Any disagreement was resolved by discussing with the senior author (Fu).

### 2.3 Data extraction and quality assessment

Two independent investigators (Chen and Liu) extracted data from each eligible study independently. The following data, if available, were recorded: the first author' last name, year of publication, country or region, study period, sample size (total patients and percentage of upstaging), patients age, tumor stage, treatment, median or mean follow-up time, and oncologic outcomes (RFS or OS or CSS). After that, HRs and corresponding 95% CIs associated with these oncologic outcomes were extracted to perform cumulative analyses.

Study quality was scored using the Newcastle Ottawa Scale (NOS), which was recommended by Cochrane Collaboration for the assessment of non-randomized studies 20. Each literature was evaluated based on the following three domains: selection, comparability and ascertainment of outcome. The total scores were added by these three domains (ranging from 0 to 9) and more scores means better methodological quality. We defined studies with scores no less than 6 were qualified to be included in the following analysis. Discrepancies between investigators were solved through consulting the senior author (Fu).

### 2.4 Statistical analysis

Pooled HR with its corresponding 95% CI was calculated to evaluate the upstaging (cT1 upstaged to pT3) on the survival of cT1 RCC patients, and HR greater than one indicated a worse prognosis in patients with postoperative pathological upstaging. Statistical heterogeneity was evaluated using Cochrane Q test and *I^2^* metrics. *I^2^*>50% indicated obvious heterogeneity among studies 21, and a random effect model was used to pool the results. Or else, a fixed effect model was applied. Besides, sensitivity analysis was performed by sequential omission of each single study to evaluate the stability of results. The risk of publication bias was assessed by visual inspection of the funnel plots, Begg's test 22 and Egger's test 23. All above statistical analyses were performed using the STATA version 12.0 (State Corporation, College Station, TX, USA). All statistical tests were two sided, and significant difference was considered when a *P* value less than 0.05.

## 3. Results

### 3.1 Search results

**Figure 1:**
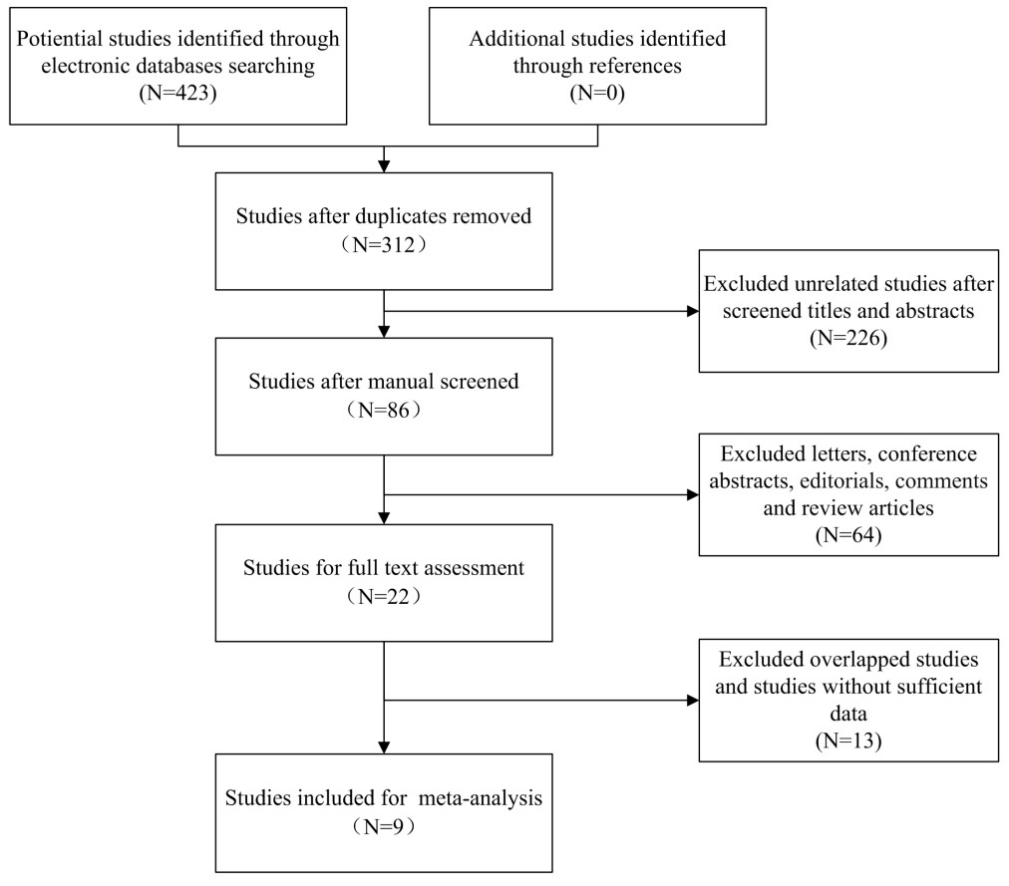
Flow chart of study selection

Our search strategy identified 423 potential relevant studies from initial literature searching. Using Endnote software, a total of 111 duplicated articles were excluded. After carefully screening titles and abstracts, 290 studies were excluded based on abovementioned inclusion criteria. The remaining 22 studies were selected for full text evaluation, in which 13 studies belonged to duplicated publication or failed to offer sufficient data (HRs with corresponding 95% CI). At last, nine studies (ten cohorts) stratified our eligibility criteria and were included in the following meta-analysis, in which the study by Srivastava et al.^24^ reported the HRs and 95% CIs of two different cohorts separately (cT1a upstaged to pT3a and cT1b upstaged to pT3a). The flowchart describing the process of literature searching is shown in Figure 1.

### 3.2 Study characteristics

The characteristics of nine included studies (ten cohorts) were summarized in Table 1. The enrolled studies all focused on the prognostic significance of pT3a upstaging in cT1 RCC and most of them were published in recent three years. These studies involved 101,505 cT1 RCC patients, of which 5,560 (5.5%) patients were up-staged to T3a after surgical treatment (conformed by pathological methods). PN as the only treatment for cT1 RCC was reported in four included studies, while others were treated with mixed therapies (PN&RN). These RCC patients came from different countries (United State, Korea and Canda). The sample size of each study ranged from 186 to 63,005 and the percentage of pT3a upstaging ranged from 3.2% to 31%. In term of follow up time, the median or mean duration period ranged from 23 to more than 60 months. Among the eligible nine studies, seven studies 12-15, 17, 25, 26 containing 10,456 patients were performed to evaluate the impact of pT3a upstaging on the RFS of cT1 RCC patients, six studies (seven cohorts) 12, 15, 24-27 containing 98,884 patients were conducted to investigate the OS and three studies (four cohorts) 12, 24, 26 containing 33,470 patients reported the CSS, respectively. Quality scores of these studies by NOS ranged from 7 to 9, which were considered adequate for the following meta-analysis.

### 3.3 Recurrence-free survival

**Figure 2:**
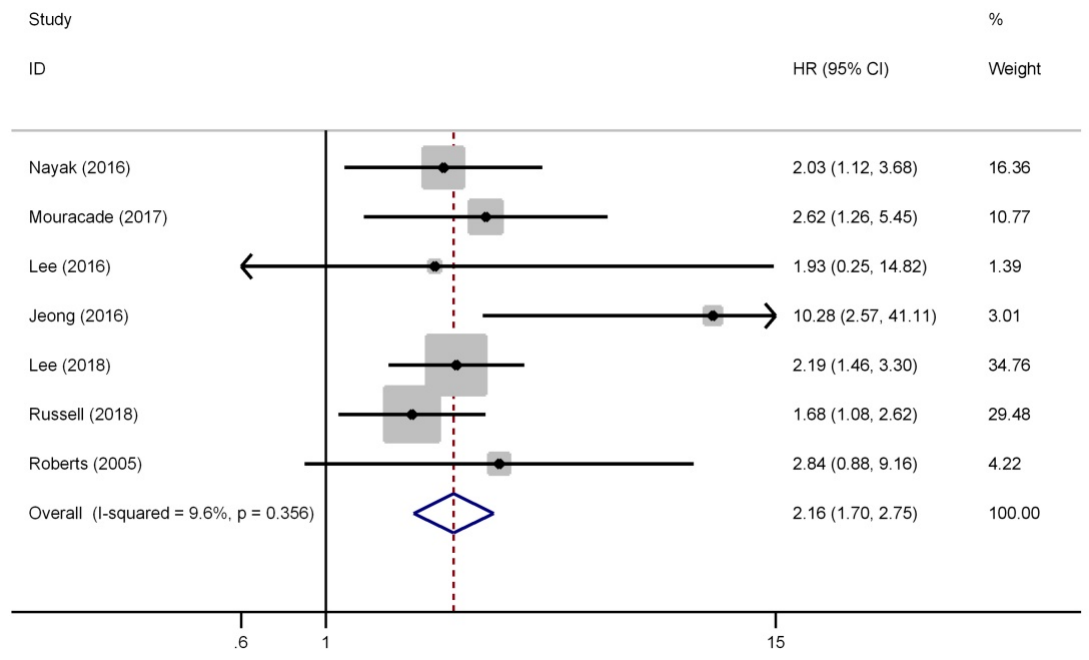
Forest plot of studies evaluating the association between pT3a upstaging and recurrence-free survival of cT1 renal cell carcinoma

There were seven studies have reported the impact of pT3a upstaging on the RFS of cT1 RCC. No evident heterogeneity existed among these studies (*I^2^*=9.6%, P=0.356), thus, a fixed effect model was used to calculate the pooled HR and its 95% CI. As presented in Figure 2, the combined results showed that the pooled HR was 2.16 and the corresponding 95% CI was 1.70-2.75 (P<0.001), which revealed that pT3a upstaging was significant associated with poorer RFS in cT1 RCC patients. Furthermore, subgroup analyses were performed by patients' ethnicity, surgical type, and analysis style. The results showed that the combined HRs estimate for RFS in Caucasian and Asian were 1.98 (P<0.001) and 2.46 (P<0.001), respectively. Besides, for patients who only underwent PN, pT3a upstaging was significant associated with poor RFS (HR=1.89, 95% CI: 1.30-2.75, P=0.001), and for those undergoing PN and RN (mixed therapies), the combined HR was 2.37 (P<0.001), which indicated that different surgical types did not affect the results. Similar findings could be found in subgroup analysis by analysis style (P<0.001).

### 3.4 Overall survival

**Figure 3:**
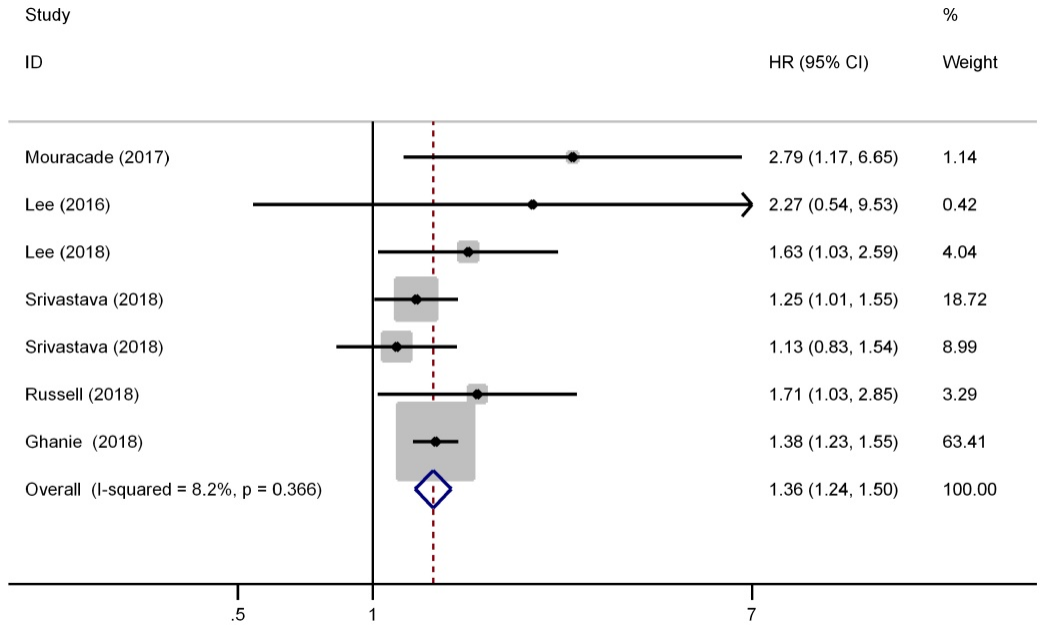
Forest plot of studies evaluating the association between pT3a upstaging and overall survival of cT1 renal cell carcinoma

There were six studies (seven cohorts) have reported the impact of pT3a upstaging on the OS of cT1 RCC. No evident inter-study heterogeneity was observed in these studies that focused on OS (*I^2^*=8.2%, P=0.366). A fixed model was applied to pool the results and the combined HR for OS was 1.36 (95% CI, 1.24-1.50, P<0.001), indicating that pT3a upstaging was associated with worse OS in patients with cT1 RCC (Figure 3). Further subgroup analysis demonstrated that pT3a upstaging was also associated with worse OS in Caucasian and Asian patients and in patients received PN or mixed therapies.

### 3.5 Cancer-specific survival

**Figure 4:**
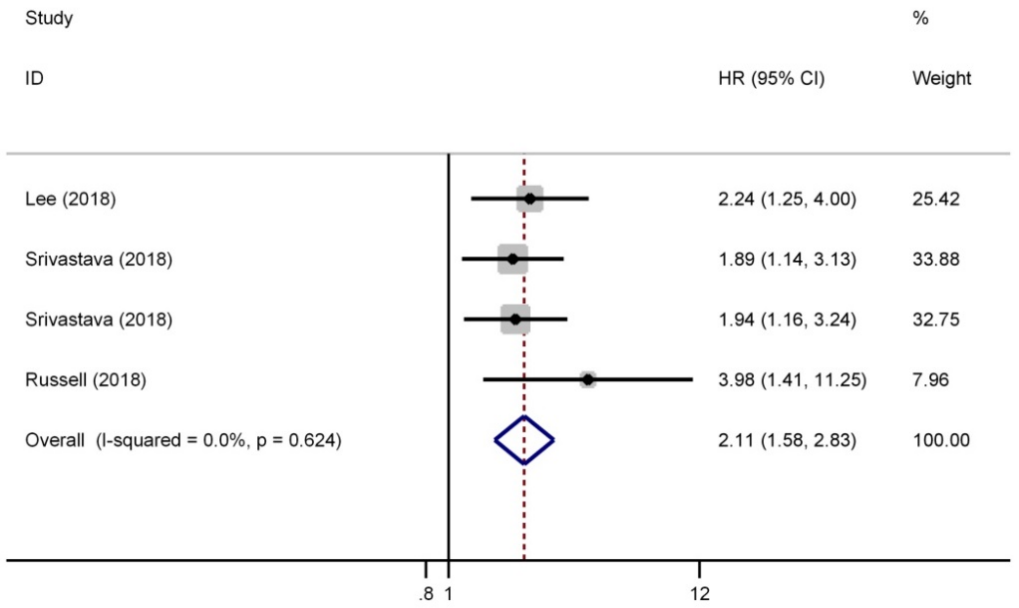
Forest plot of studies evaluating the association between pT3a upstaging and cancer-specific survival of cT1 renal cell carcinoma

There were three studies (four cohorts) have reported the impact of pT3a upstaging on the CSS of cT1 RCC. As shown in Figure 4, a fixed effect model was selected because there was no evident heterogeneity among the four studies (*I^2^*=0.0%, P=0.624). The pooled results showed that pT3a upstaging had a negative impact on the CSS of cT1 RCC patients who received surgical treatment (HR=2.11, 95% CI: 1.58-2.83, P<0.001).

### 3.6 Sensitivity analysis

**Figure 5:**
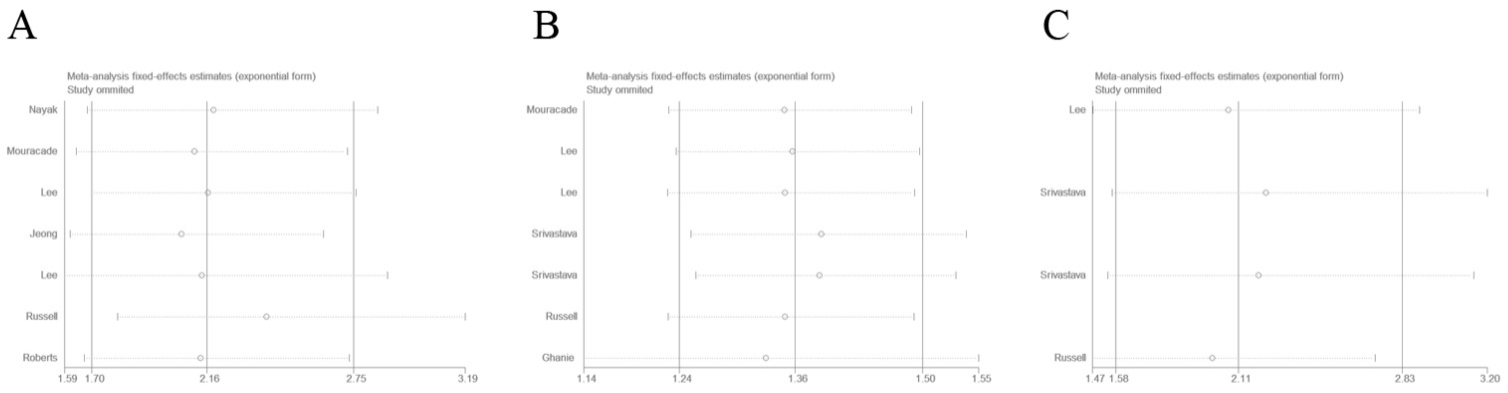
** Sensitive analysis of included studies.** A: recurrence-free survival; B: overall survival; C: cancer-specific survival

Sensitivity analysis was conducted by sequential omission of each single study. As shown in Figure 5, the results showed that the merged HRs of RFS, OS, and CSS did not significantly changed, which confirmed the credibility of our outcomes.

### 3.7 Publication bias

**Figure 6:**
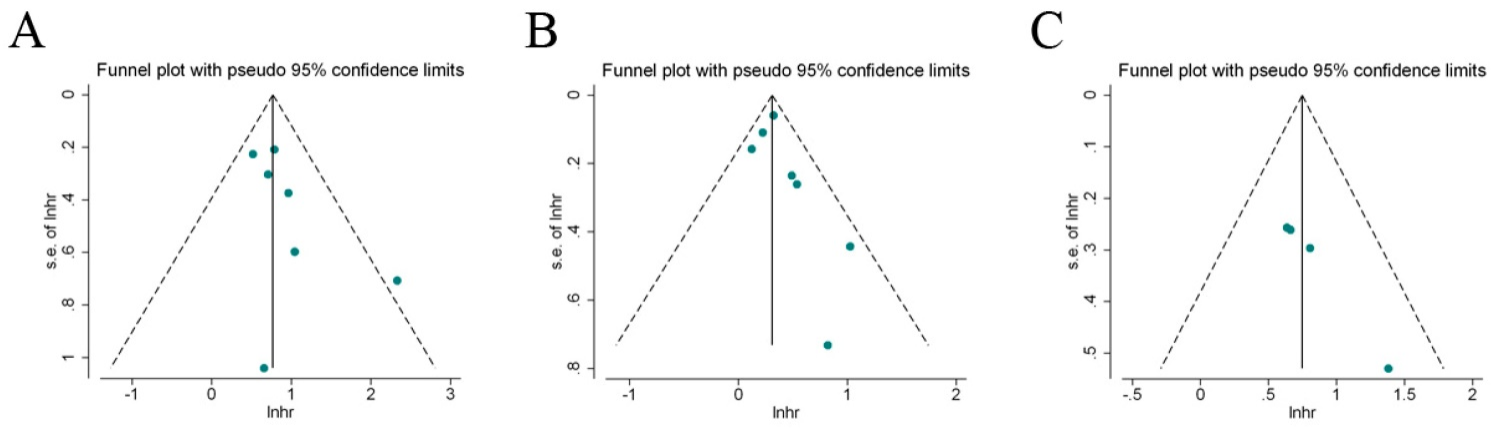
** Funnel plots for the evaluation of potential publication bias.** A: recurrence-free survival; B: overall survival; C: cancer-specific survival

Begg's test and Egger's test, as well as visual inspection of funnel plot were performed to estimate the publication bias in our meta-analysis. Figure 6 indicated that these included studies had no evident asymmetry in the funnel plots (RFS, OS, and CSS). Besides, the results by begg's test and Egger's test for the enrolled studies assessing the survival outcomes were P_begg_=0.386, P_egger_=0.152 (RFS); P_begg_=0.230, P_egger_=0.230 (OS); P_begg_=0.089, P_egger_=0.078 (CSS), respectively. Therefore, the abovementioned evidences revealed a low probability of publication bias in the present meta-analysis.

## 4. Discussion

Epidemiological data suggested that the incidence of RCC has steadily increased in recent years. The increase might be partially attributed to the widespread use of non-invasive imaging techniques, thereby resulting in the early detection of small renal tumors. Currently, The TNM staging system remains the most widely accepted system for treatment determination 28. However, renal sinus fat or perirenal fat invasion or renal vein thrombosis may be missed by perioperative CT 10, 11, and thus RCC may upstage from cT1 to pT3a after surgery. This interesting clinical issue has attracted extensive attention and has been widely debated. Previous reports regarding the prognostic significance of incidental pT3 upstaging in cT1 RCC remain conflicting and controversial. Thus, we systemically review the relevant published studies and conducted a standard meta-analysis to clarify the prognostic value of postoperative pT3 upstaging in patients with cT1 RCC.

In the present research, nine studies (ten cohorts) were eligible based on the inclusion criteria. HRs of cumulative survival (RFS, OS and CSS) were summarized quantitatively by standard meta-analysis techniques. The combined results demonstrated that postoperative pT3a upstaging was significantly associated with poor RFS in patients with cT1 RCC. Similar results were found in analyses on OS and CSS. Notably, in the subgroup analysis, for patients receiving PN or mixed nephrectomy (PN and RN), pT3a upstaging from cT1 still had a negative impact on the survival, thereby indicating that different surgical types did not influence the negative prognostic significance of pT3a upstaging.

Currently, PN has been the standard treatment for T1 RCC, because it preserves renal function that relates to reduced renal and cardiovascular complications and provides better overall survival than RN. However, whether cT1 RCCs with postoperative pT3a upstaging treated by PN have equivalent clinical survival outcomes compared with those treated by RN or not is not clear, and this interesting issue deserves to be discussed. Shah et al. 29 retrospectively reviewed the records of 1,250 patients who underwent PN or RN for cT1 RCC and T3a upstaging was noted in 140 patients (11%). Further subgroup analysis among upstaged T3a cases demonstrated that the risk of relapse in PN is higher than that in RN. A similar study by Jeong et al. 13 contradicted Shah's findings and revealed no difference in RFS between PN and RN in RCC patients with incidental pT3a upstaging. Besides, Weight et al. 30 analyzed the OS and CSS among patients who underwent RN or PN with cT1 and pT3 upstaging and found equivalent survivals in PN and RN groups. Moreover, Hansen et al.^31^ suggested results similar to those of Weight's study regarding the CSS among RCC patients with pT3a disease. Previous relevant studies provided inconsistent findings but the majority agreed that PN could provide at least equivocal oncological outcomes in patients with cT1/pT3a RCC. Our results showed that the incidence of upstaging was relatively unusual (5.5%). Thus, the majority of patients will still benefit from PN. Therefore, clinicians should not avoid PN because of concerns regarding upstaging, although cautious follow-up is warranted in cases with upstaging.

Our meta-analysis with large samples confirmed that patients with pathological T3a-upstaged cT1 RCC had worse clinical oncological outcomes than patients with non-upstaged cT1 RCC. Therefore, the predictors of upstaging, which might greatly aid in the preoperative counseling of patients regarding the risk of pT3a, must be considered before RCC treatment. Thus far, a series of studies has focused on this topic. Lee et al. 12 found that patient age, tumor diameter, and hilar location were significantly associated with a risk of pathological upstaging. Nayak et al. 14 showed that increasing age and tumor size were independent predictors and found that Fuhrman grade, when assessed preoperatively by a biopsy, is another predictor and might be helpful in determining the probability of upstaging. This finding is consistent with consistent with the findings of Jeongs' 13 and Ghanie's 27. Furthermore, a high R.E.N.A.L. nephrometry score has been recognized by several studies to be an important factor associated with tumor up-staging 25, 32, 33. These factors might predict upstaging preoperatively and provide valuable adjunct information about risk stratification. Furthermore, aggressive management strategies, such as removing fat with the tumor and avoiding take an enucleation approach, can be offered to patients who are most likely to be upstaged.

Postoperative T3a upstaging has a significantly negative impact on survival outcomes, especially the RFS, of patients with cT1 RCC. Thus, these high-risk patients should be closely monitored by clinicians and should receive close follow-up. Moreover, the utility of adjuvant systemic therapies in patients with increased risk of recurrence has attracted some attention and has been discussed in recent years 34-37. A randomized, double-blind, Phase III trial (S-TRAC) 34 was performed to determine the efficacy of sunitinib in patients with loco-regional RCC at high risk for tumor recurrence after nephrectomy. The results showed that the median duration of disease-free survival was significantly longer in the sunitinib group than in the placebo group. Another Phase III Trial (PROTECT) 35 demonstrated increased disease-free survival outcome in locally advanced renal cell carcinoma patients receiving adjuvant pazopanib. These studies emphasize the potential benefit of target therapy in select patients, and further studies specifically designed to evaluate patients with cT1 and pT3a upstaging are required to confirm the efficacy.

To the best of our knowledge, our study is the first standard meta-analysis that assessed the association between pT3a upstaging and survivals of cT1 RCC. Our systematic review of nine published studies (ten cohorts) including more than 10,000 samples indicated that the postoperative pT3a upstaging of cT1 RCC was unusual (5.5%). However, its prognostic value should not be ignored because the accurate evaluation of prognosis, especially after surgery, is highly important for the planning of surveillance program and the following relevant adjuvant therapy. Our results quantified the impact of pT3a upstaging to be a negative prognostic factor. However, several limitations of this study must also be acknowledged. First, most of these eligible studies in this systematic review were retrospective studies. Second, among the enrolled studies, some studies did not provide HRs directly and were calculated using the methods reported by Tierney et al. 19 These calculated HRs might not be as dependable as those retrieved directly from reported results. Third, the influence of different subtypes of T3a upstaging (renal sinus fat or perirenal fat invasion or renal vein thrombosis) on survival outcomes was not discussed owing to insufficient data. Besides, only four cohorts investigated the CSS of cT1 RCC by a comprehensive literature search, thereby probably increasing the risk of random error. Therefore, additional well-designed studies are still required to further confirm our finding. Finally, the unavoidable limitations exist. The results of all meta-analysis were affected by the quality of component studies. The situation wherein studies with insignificant results are more difficult to publish in journals than those with statistically significant results may compromise the validity of the meta-analysis 38.

## 5. Conclusions

In summary, our meta-analysis of current available evidences strongly indicates that postoperative pT3a upstaging is significantly associated with poor RFS, OS, and CSS in patients with cT1 RCC. For patients with pT3a upstaging, close monitoring and follow ups are required. Several promising upstaging predictors can provide valuable information to clinicians, and adjuvant target therapy might benefit these high-risk patients but still require further evaluation.

## Figures and Tables

**Table 1:** Characteristics of eligible studies in the meta-analysis.

Author	Year	Country	Study period	RCC Patients	Upstage to pT3a	Age (years)	Treatment	Follow up (median)	Outcome	Analysis	Quality score
Nayak	2016	Canada	2009-2015	1448 cT1	134(9%)	Median 59.0	PN & RN	23 months	RFS	Multi	9
Mouracade	2017	USA	2007-2015	1042 cT1	113(10.8%)	Median 60.0	PN	35 months	RFS, OS	Uni	8
Lee	2016	Korea	1997-2014	1367 cT1a	43(3.2%)	Median 53.9	PN	54 months	RFS, OS	Uni	7
Jeong	2016	Korea	2001-2013	987 cT1	91 (9.2%)	Mean 54.9	PN & RN	48.5 months*	RFS	Uni	7
Lee	2018	Korea	1997-2016	3431 cT1	215(6.3%)	Median 55.0	PN & RN	39.0 months	RFS, OS, CSS	Multi	9
Srivastava	2018	USA (SEER)	1998-2013	23246 cT1a 4798 cT1b	976(4.2%) 456(9.5%)	Median 60.0	PN	40.0 months	OS, CSS	Multi	8
Russell	2018	USA	1995-2015	1995 cT1	95(4.8%)	Median 61.5	PN	38.2 months	RFS, CSS, OS	Uni	9
Ghanie	2018	USA(NCDB)	2010-2013	63005 cT1	3380(5.4%)	Mean 60.1	PN & RN	>5 years	OS	Multi	7
Roberts	2005	USA	1990-1999	186 cT1	57(31%)	NA	PN & RN	52.6 months*	RFS	Uni	7

Abbreviations: RCC: renal cell carcinoma; RFS: recurrence-free survival; OS: overall survival; CSS: cancer-specifi survival; PN: partial nephrectomy; RN: radical nephrectomy; NA: not available.

**Table 2:** Subgroup analysis of the studies reporting the association of pT3a upstaging and RFS/OS of cT1 RCC.

Subgroup	Studies	Pooled HR	95% CI	P	Heterogeneity (*I*^2^)
**RFS**					
**Ethnicity**					
Caucasian	4	1.98	1.46-2.70	P<0.001	0.0%
Asian	3	2.46	1.68-3.61	P<0.001	55.0%
**Surgical type**					
PN	3	1.89	1.30-2.75	P=0.001	0.0%
PN & RN	4	2.37	1.73-3.25	P<0.001	37.4%
**Analysis type**					
univariable analysis	5	2.18	1.70-2.75	P<0.001	39.3%
multivariable analysis	2	2.14	1.53-3.00	P<0.001	0.0%
**OS**					
**Ethnicity**					
Caucasian	5	1.35	1.23-1.48	P<0.001	26.3%
Asian	2	1.68	1.09-2.61	P=0.020	0.0%
**Surgical type**					
PN	5	1.30	1.11-1.53	P=0.002	28.3%
PN & RN	2	1.39	1.25-1.56	P<0.001	0.0%
**Analysis type**					
univariable analysis	3	1.97	1.29-2.99	P=0.002	0.0%
multivariable analysis	4	1.34	1.22-1.47	P<0.001	0.0%

Abbreviations: RFS: recurrence-free survival; OS: overall survival; RCC: renal cell carcinoma; HR: hazard ratio; CI: confidence interval; PN: partial nephrectomy; RN: radical nephrectomy.

## References

[1] Miller KD, Siegel RL, Lin CC (2016). Cancer treatment and survivorship statistics, 2016. CA Cancer J Clin.

[2] Chow WH, Dong LM, Devesa SS (2010). Epidemiology and risk factors for kidney cancer. Nat Rev Urol.

[3] Siegel RL, Miller KD, Jemal A (2018). Cancer statistics, 2018. CA Cancer J Clin.

[4] Murai M, Oya M (2004). Renal cell carcinoma: etiology, incidence and epidemiology. Curr Opin Urol.

[5] Patard JJ, Tazi H, Bensalah K (2004). The changing evolution of renal tumours: a single center experience over a two-decade period. Eur Urol.

[6] Thompson RH, Siddiqui S, Lohse CM (2009). Partial versus radical nephrectomy for 4 to 7 cm renal cortical tumors. J Urol.

[7] Kreshover JE, Richstone L, Kavoussi LR (2013). Renal cell recurrence for T1 tumors after laparoscopic partial nephrectomy. J Endourol.

[8] Hennessey DB, Wei G, Moon D (2018). Strategies for success: a multi-institutional study on robot-assisted partial nephrectomy for complex renal lesions. BJU Int.

[9] Borghesi M, Schiavina R, Gan M (2013). Expanding utilization of robotic partial nephrectomy for clinical T1b and complex T1a renal masses. World J Urol.

[10] Sokhi HK, Mok WY, Patel U (2015). Stage T3a renal cell carcinoma: staging accuracy of CT for sinus fat, perinephric fat or renal vein invasion. Br J Radiol.

[11] Tsili AC, Goussia AC, Baltogiannis D (2013). Perirenal fat invasion on renal cell carcinoma: evaluation with multidetector computed tomography-multivariate analysis. J Comput Assist Tomogr.

[12] Lee H, Lee M, Lee SE (2018). Outcomes of pathologic stage T3a renal cell carcinoma up-staged from small renal tumor: emphasis on partial nephrectomy. BMC Cancer.

[13] Jeong SH, Kim JK, Park J (2016). Pathological T3a Upstaging of Clinical T1 Renal Cell Carcinoma: Outcomes According to Surgical Technique and Predictors of Upstaging. PLoS One.

[14] Nayak JG, Patel P, Saarela O (2016). Pathological Upstaging of Clinical T1 to Pathological T3a Renal Cell Carcinoma: A Multi-institutional Analysis of Short-term Outcomes. Urology.

[15] Lee C, You D, Yoo S (2016). Oncological outcomes of patients with incidental pathological T3a stage small renal cell carcinoma after partial nephrectomy. Journal of Cancer Research and Clinical Oncology.

[16] Ramaswamy K, Kheterpal E, Pham H (2015). Significance of Pathologic T3a Upstaging in Clinical T1 Renal Masses Undergoing Nephrectomy. Clin Genitourin Cancer.

[17] Roberts WW, Bhayani SB, Allaf ME (2005). Pathological stage does not alter the prognosis for renal lesions determined to be stage T1 by computerized tomography. J Urol.

[18] Moher D, Liberati A, Tetzlaff J (2010). Preferred reporting items for systematic reviews and meta-analyses: the PRISMA statement. Int J Surg.

[19] Tierney JF, Stewart LA, Ghersi D (2007). Practical methods for incorporating summary time-to-event data into meta-analysis. Trials.

[20] Stang A (2010). Critical evaluation of the Newcastle-Ottawa scale for the assessment of the quality of nonrandomized studies in meta-analyses. Eur J Epidemiol.

[21] Higgins JP, Thompson SG, Deeks JJ (2003). Measuring inconsistency in meta-analyses. BMJ.

[22] Begg CB, Mazumdar M (1994). Operating characteristics of a rank correlation test for publication bias. Biometrics.

[23] Egger M, Davey Smith G, Schneider M (1997). Bias in meta-analysis detected by a simple, graphical test. BMJ.

[24] Srivastava A, Patel HD, Joice GA (2018). Incidence of T3a up-staging and survival after partial nephrectomy: Size-stratified rates and implications for prognosis. Urol Oncol.

[25] Mouracade P, Kara O, Dagenais J (2017). Perioperative morbidity, oncological outcomes and predictors of pT3a upstaging for patients undergoing partial nephrectomy for cT1 tumors. World J Urol.

[26] Russell CM, Lebastchi AH, Chipollini J (2018). Multi-institutional Survival Analysis of Incidental Pathologic T3a Upstaging in Clinical T1 Renal Cell Carcinoma Following Partial Nephrectomy. Urology.

[27] Ghanie A, Formica MK, Wang D (2018). Pathological upstaging of clinical T1 renal cell carcinoma: an analysis of 115,835 patients from National Cancer Data Base, 2004-2013. Int Urol Nephrol.

[28] Ficarra V, Galfano A, Mancini M (2007). TNM staging system for renal-cell carcinoma: current status and future perspectives. Lancet Oncol.

[29] Shah PH, Moreira DM, Patel VR (2017). Partial Nephrectomy is Associated with Higher Risk of Relapse Compared with Radical Nephrectomy for Clinical Stage T1 Renal Cell Carcinoma Pathologically Upstaged to T3a. J Urol.

[30] Weight CJ, Lythgoe C, Unnikrishnan R (2011). Partial nephrectomy does not compromise survival in patients with pathologic upstaging to pT2/pT3 or high-grade renal tumors compared with radical nephrectomy. Urology.

[31] Hansen J, Sun M, Bianchi M (2012). Assessment of cancer control outcomes in patients with high-risk renal cell carcinoma treated with partial nephrectomy. Urology.

[32] Gorin MA, Ball MW, Pierorazio PM (2013). Outcomes and predictors of clinical T1 to pathological T3a tumor up-staging after robotic partial nephrectomy: a multi-institutional analysis. J Urol.

[33] Tay MH, Thamboo TP, Wu FM (2014). High R.E.N.A.L. Nephrometry scores are associated with pathologic upstaging of clinical T1 renal-cell carcinomas in radical nephrectomy specimens: implications for nephron-sparing surgery. J Endourol.

[34] Ravaud A, Motzer RJ, Pandha HS (2016). Adjuvant Sunitinib in High-Risk Renal-Cell Carcinoma after Nephrectomy. N Engl J Med.

[35] Motzer RJ, Haas NB, Donskov F (2017). Randomized Phase III Trial of Adjuvant Pazopanib Versus Placebo After Nephrectomy in Patients With Localized or Locally Advanced Renal Cell Carcinoma. J Clin Oncol.

[36] Haas NB, Manola J, Uzzo RG (2016). Adjuvant sunitinib or sorafenib for high-risk, non-metastatic renal-cell carcinoma (ECOG-ACRIN E2805): a double-blind, placebo-controlled, randomised, phase 3 trial. Lancet.

[37] Haas NB, Manola J, Dutcher JP (2017). Adjuvant Treatment for High-Risk Clear Cell Renal Cancer: Updated Results of a High-Risk Subset of the ASSURE Randomized Trial. JAMA Oncol.

[38] Sutton AJ, Song F, Gilbody SM (2000). Modelling publication bias in meta-analysis: a review. Stat Methods Med Res.

